# Medical Association of Georgia

**Published:** 1880-05-20

**Authors:** 


### *AMERICAN MEDICAL ASSOCIATION.*

### *MEDICAL ASSOCIATION OF GEORGIA.*

We glean from the Augusta papers the following facts in relation to the Georgia Medical Association, which met in Augusta, Georgia, on April 21st.

The address of welcome was delivered by Prof. H. F. Campbell, of Augusta, in his peculiar eloquent and happy style.

The annual address was delivered by Prof. Joseph Eve, the President of the Association. It was an able and interesting address, and evinces that age and hard service in the profession have not detracted from the power and eloquence of this honored veteran in our ranks.

We regret that we have not space to publish this address entire.

A large number of members were enrolled and quite a number of new members were initiated.

The Committee to present the claims of the late Dr. Crawford W. Long, as the discoverer of Anæsthesia, before the American Medical Association was granted further time.

On the second day of the Association, Dr. W. P. Nicolson, Prof. of Anatomy in the Southern Medical College, Atlanta, was introduced as the Delegate from the Virginia State Medical Society, and was also admitted to membership of the Georgia Association.

A number of interesting papers were presented.

Dr. DeSaussure Ford read a voluntary paper from Dr. L. D. Ford, on "Organic Affinity and Vital Selection."

Dr. Thos. . Wright presented a paper on a case of incised wound of the elbow.

Dr. A. Sibley Campbell presented a voluntary report of surgical causes.

Dr. Eugene Foster read a full and very able paper on the epidemic of yellow fever in Augusta. This paper was listened to with very deep attention by the Association. Dr. Foster took the ground that yellow fever can originate in a city, the proper conditions existing to produce it. This was combatted by Dr. Henry F. Campbell, who held that it was impossible for yellow fever to originate in Augusta. It must be imported. Trash piles might be piled up mountain high, malarial fevers might prevail, but there could be no yellow fever unless the germs were brought here from infected districts. These germs might be brought in trunks, in clothing, or in the hair. A person might come from an infected district, change his clothing at the quarantine station, twenty miles away, have his hair washed with carbolic acid if need be, and there could then be no possible danger to the people of the city, by his coming into it, even though the black vomit was gushing from his mouth. He denounced the present personal quarantine system as cruel and horrible, and said if it was persisted in, it would result in insurrection.

The discussion on the paper occupied nearly the entire day and was interesting and instructive.

The Chairman announced the following list of delegates to the American Medical Association at its session in New York, next month :

Henry Gaithen, Oxford; Thos. R. Wright, Augusta; Robt. Battey, Rome; J. P. Logan, Atlanta; J. B. S. Holmes, Rome; H. F. Campbell, Augusta; Wm. F. Hitt, Macon; E. W. Alfriend, Albany; W. J. Howell, Bainbridge; T. M. McIntosh, Thomasville; J. C. LeHardy, Savannah; T. S. Powell, Atlanta; F. A. Standford, Columbus; A. G. Whitehead, Waynesboro; Thos. Raines, Atlanta; J. W. Bailey, Gainesville; Jno. G. Thomas, Savannah; DeSaussure Ford, Augusta; A. W. Griggs, West Point; G. F. Cooper, Americus; Wm. O'Daniel, Bullard; C. H. Hall, Macon; K. P. Moore, Forsyth; A. Sibley Campbell, Augusta; W. B. Wells, Red Clay.

The Committee on Nominations reported the following nominations for officers for the ensuing year :

*For President*—Dr. J. C. LeHardy, of Savannah.

*First Vice-President*—E. W. Alfriend, of Albany.

*Second Vice-President*—W. B. Wells, of Red Clay.

*Censor*—E. L. Connelly.

*Committee on Publications*—Drs. Jas. B. Baird, T. J. Johnson, W. S. Armstrong, W. T. Goldsmith, K. P. Moore.

*Committee on Necrology*—K. P. Moore, A. S. Campbell, T. S. Hopkins, J. B. S. Holmes, C. B. Leiner.

Prof. H. F. Scott, of the Southern Medical College, was elected to deliver the annual oration at the next meeting of the Association.

The report was unanimously adopted.

The Secretary and the Treasurer are elected every five years. Dr. Jas. B. Baird, of Atlanta, is the Secretary, and Dr. K. P. Moore, of Forsyth, the Treasurer of the Association.

The last evening an eloquent and interesting address was delivered at Masonic Hall by Dr. W. S. Kendrick, of Atlanta. At ten o'clock the Association and their invited guests proceeded to the Planters' Hotel, where an elegant banquet was spread on three long tables. After a Blessing was asked by Rev. W. B. Walker, the substantials and delicacies, with a liberal accompaniment of punch and champagne, were discussed to the satisfaction of all present. There were no regular toasts, but several impromptu sentiments were proposed, among them the following :

The Medical Association of Georgia—Responded to by Dr. Doster, of Blakely.

The Nestor of the Medical Profession of Georgia—Responded to by Dr. L. D. Ford.

The Southern Medical College—Responded to, in a happy manner, by Dr. Nicolson.

The Atlanta Medical College—Responded to by Dr. J. Thad. Johnson.

The Legal Profession—Responded to by Gen. M. W. Gary. Gen. Gary was very happy in his remarks, and loudly applauded.

The Secretary—Responded to by Dr. Baird.

The Committee of Arrangements—Responded to by Dr. A. Sibley Campbell.

The Press—Responded to by Mr. James R. Randall.

The Clergy—Responded to by Rev. Father McNally.

The next meeting of the Association will be held at Thomasville, April, 1881.

